# Functional Outcome Prediction in Young Adults With Mental Health Symptoms Using Machine Learning and Large Language Models: Longitudinal Observational Study

**DOI:** 10.2196/84424

**Published:** 2026-06-22

**Authors:** Pavol Mikolas, Fabian Huth, Kyra Bröckel-Bundt, Christina Berndt, Julia Martini, Birgit Maicher, Michael Marxen, Falk Gerrik Verhees, Paula Marie Henneberg, Christoph Vogelbacher, Andreas Jansen, Tilo Kircher, Irina Falkenberg, Florian Thomas-Odenthal, Martin Lambert, Vivien Kraft, Gregor Leicht, Christoph Mulert, Andreas J Fallgatter, Thomas Ethofer, Anne Rau, Karolina Leopold, Andreas Bechdolf, Reif Andreas, Silke Matura, Jonathan Repple, Felix Bermpohl, Jana Fiebig, Thomas Stamm, Christoph U Correll, Georg Juckel, Vera Flasbeck, Isabella C Wiest, Jakob N Kather, Udo Dannlowski, Eva Mennigen, Philipp Ritter, Michael Bauer, Andrea Pfennig

**Affiliations:** 1 Department of Psychiatry and Psychotherapy, Carl Gustav Carus University Hospital, TUD Dresden University of Technology, Dresden, Germany; 2 Department of Psychiatry and Psychotherapy, Jena University Hospital, Philosophenweg 3, Jena, Thuringia, 07743, Germany, 49 36419300; 3 Department of Psychiatry, University of Marburg, Marburg, Germany; 4 Core-Facility Brain Imaging, Faculty of Medicine, University of Marburg, Marburg, Germany; 5 Center for Mind, Brain and Behavior (CMBB), University of Marburg and Justus Liebig University Giessen, Marburg and Giessen, Germany; 6 Department of Psychiatry and Psychotherapy, University Medical Center Hamburg-Eppendorf, Hamburg, Germany; 7 Centre for Psychiatry, Justus-Liebig University Giessen, Giessen, Germany; 8 Tübingen Center for Mental Health, Department of Psychiatry, University of Tübingen, Tübingen, Baden-Wurttemberg, Germany; 9 Partner Site Tübingen, Deutsches Zentrum für Psychische Gesundheit, Tübingen, Germany; 10 Biomedical Magnetic Resonance, University of Tübingen, Tuebingen, Baden-Wurttemberg, Germany; 11 Vivantes Hospital Am Urban and Vivantes Hospital Im Friedrichshain, Department of Psychiatry, Psychotherapy and Psychosomatic Medicine, Charité-Universitätsmedizin Berlin, Berlin, Germany; 12 Department of Psychiatry, Psychosomatic Medicine and Psychotherapy, University Hospital, Goethe University Frankfurt, Frankfurt, Germany; 13 Cooperative Brain Imaging Center - CoBIC, Goethe University Frankfurt, Frankfurt, Hesse, Germany; 14 Institute for Translational Psychiatry, University of Münster, Münster, North Rhine-Westphalia, Germany; 15 Charité Campus Mitte, Department of Psychiatry and Psychotherapy, Charité University Medicine, Berlin, Germany; 16 Department of Clinical Psychiatry and Psychotherapy, Brandenburg Medical School Theodor Fontane, Neuruppin, Brandenburg, Germany; 17 Department of Child and Adolescent Psychiatry, Charité Universitätsmedizin Berlin, Berlin, Germany; 18 Department of Psychiatry, Northwell Health, Zucker Hillside Hospital, New York, NY, United States; 19 Department of Psychiatry and Molecular Medicine, Donald and Barbara Zucker School of Medicine at Hofstra/Northwell, Hempstead, NY, United States; 20 Department of Psychiatry, Psychotherapy and Preventive Medicine, LWL University Hospital, Ruhr-University, Bochum, Germany; 21 Else Kroener Fresenius Center for Digital Health, TUD Dresden University of Technology, Dresden, Germany; 22 Institute for Translational Psychiatry, University of Münster, Münster, Germany; 23 Department of Psychiatry, Medical School and University Medical Center OWL, Protestant Hospital of the Bethel Foundation, Bielefeld University, Bielefeld, Germany; 24 German Center for Mental Health (DZPG), Jena Magdeburg Halle, Germany; 25 Center for Intervention and Research on Adaptive and Maladaptive Brain Circuits Underlying Mental Health (C-I-R-C), Jena Magdeburg Halle, Germany

**Keywords:** machine learning, magnetic resonance imaging, longitudinal studies, natural language processing, prognosis, young adult, mental disorders, neuroimaging

## Abstract

**Background:**

Functional impairments associated with mental health conditions are on the rise. Predicting functional outcomes may improve the targeting of preventive interventions. While prognostic models have primarily focused on psychosis, early recognition services require a transdiagnostic approach.

**Objective:**

This study aimed to predict global functioning within a 2-year follow-up using baseline clinical and structural magnetic resonance imaging (MRI) data in a population-based sample of young, help-seeking individuals presenting with affective and anxiety symptoms as well as attention-deficit hyperactivity disorder.

**Methods:**

We classified 357 help-seeking individuals aged 18‐35 years recruited from 9 sites as “impaired” (Global Assessment of Functioning [GAF] ≤60; n=228) or “nonimpaired” (GAF>60; n=129) at year 1 and/or year 2 follow-up. GAF classification group status at follow-up was predicted using linear support vector machine (SVM), decision tree, and large language model (LLM) Llama-3 using clinical assessments and/or structural MRI. Leave-one-site-out (SVM) or external sample (LLM) was used for validation.

**Results:**

SVM achieved balanced accuracy of 69.2% using clinical features only. Items related to baseline occupational functioning, interpersonal relationships, cognitive functioning, psychotic and affective symptoms, as well as the presence of anxiety disorder, were most predictive. The decision tree further reduced the feature set to 5 predictive items, achieving balanced accuracy of 76.6%. Although amygdala and hippocampal subregions achieved balanced accuracy of 57.1%, structural MRI did not improve the overall prediction. Llama-3 performed comparably well to SVM (balanced accuracy of 72.6%).

**Conclusions:**

Machine learning demonstrated good performance in predicting global functioning. Interestingly, the out-of-the-box LLM performed comparably well without being trained or fine-tuned, highlighting the potential of leveraging free-text data for mental health prognosis.

## Introduction

Functional impairments due to mental health conditions have been on the rise. For example, in Germany, the proportion of early retirements due to mental disorders increased from 24% to 42% between 2000 and 2022 [1]. According to the World Health Organization, mental disorders accounted for 16% of disability-adjusted life years (totaling 418 million disability-adjusted life years) worldwide in 2019 [2]. Improving early risk stratification and predicting functional outcomes may enhance the targeting of preventive measures, ultimately mitigating the long-term negative impact. Machine learning methods and large multicenter datasets have facilitated the development and validation of predictive models that incorporate not only clinical variables but also biological data, such as magnetic resonance imaging (MRI). Recently, large language models (LLMs), probabilistic transformer-based models originally developed to predict text sequences [3], have been increasingly explored for their potential in clinical applications. LLMs outperformed human experts on a range of medical diagnostic and academic tasks [4,5]. In our previous work, we compared the LLM performance to expert ratings while assessing medical text for suicidality, achieving an accuracy of 87.5% [6]. The next logical step—the ability to predict clinical outcomes—has rarely been tested. LLMs have been effective in predicting hospital readmission due to a general medical condition [7], admission to an intensive care unit [8], or intentional self-harm [9]. Given the fact that mental health care relies heavily on unstructured textual data, the potential of LLMs remains underused.

Global assessment tools, such as the Global Assessment of Functioning (GAF) [10], are among the most commonly used instruments for functional outcomes [11]. Prognostic studies have mainly focused on psychosis. In individuals at clinical high risk for psychosis, the inclusion of structural MRI data significantly enhanced the prediction of 1-year functional outcomes, achieving clinically meaningful balanced accuracy exceeding 80% [12]. In first-episode psychosis, demographic and baseline clinical data predicted GAF at 12-month follow-up with a balanced accuracy of 72% [13]. In a sample of individuals with psychosis of varying illness duration, baseline clinical data predicted GAF at 3-year and 6-year follow-ups with accuracies ranging from 63.5% to 67.6% [14]. Additionally, clustering analysis of baseline cognitive profiles identified distinct patient subgroups within first-episode psychosis, who exhibited low clinical functioning at 6- and 12-month follow-ups [15]. While psychosis prediction remains a prominent objective, the nonspecificity of prodromal symptoms and relatively low transition rates underscore the necessity for early recognition services to use a transdiagnostic approach [16]. We aimed to predict functional outcomes within a 2-year follow-up in young, help-seeking individuals with predominantly affective and anxiety symptoms, as well as attention-deficit hyperactivity disorder (ADHD), who were recruited within the Early-BipoLife study—a population-based study on bipolar risk. Along with broad baseline clinical characteristics, we explored the potential of baseline structural MRI data to improve prediction accuracy. We used data-driven feature selection and leave-one-site-out cross-validation (CV) to ensure generalizability over multiple sites. Finally, we used a locally hosted, general-purpose LLM to estimate the outcome based on engineered clinical notes derived from the baseline data. To the best of our knowledge, this is the first study to use LLMs to predict global functioning at follow-up in individuals presenting with mental health symptoms.

## Methods

### Participants

Early-BipoLife [17] is a multicenter, prospective, naturalistic study of participants aged 15‐35 years recruited between 2015 and 2019 and assessed over a period of at least 2 years. Help-seeking youth and young adults consulting early detection centers or individuals presenting with at least one of the proposed risk factors for bipolar disorder [17,18] (refer to Section 1 in Multimedia Appendix 1 for inclusion and exclusion criteria) as well as inpatients and outpatients with depressive syndrome or ADHD were recruited at 9 sites. For detailed data collection procedures, see Pfennig et al [17], Martini et al [19], and Section 1 in Multimedia Appendix 1. Comprehensive assessments were performed after 12 and 24 months. All participants received state-of-the-art counseling and treatment. Of the 918 participants who completed the 2-year follow-up, 31 (3.17%) participants transitioned to bipolar disorder [19]. In total, 313 opted to receive a baseline MRI. Given the relatively low transition rates and the absence of a focus on predicting bipolar disorder, this sample is well-suited for predicting functional outcome using a transdiagnostic approach.

### Baseline and Follow-Up Assessments

Following clinical assessments were included in the analysis of functional outcome: age, sex, medication (none or any current psychotropic medication), contact to mental health services present or past, migration status (positive when person itself, father or mother were not born in Germany and as negative if all 3 were born in Germany), first-degree relatives for bipolar disorder, *DSM-IV* (*Diagnostic and Statistical Manual of Mental Disorders* [Fourth Edition]) diagnoses and substance use (SKID-I [Structured Clinical Interview for *DSM-IV* Axis I Disorders]) [20], depressive symptoms (Inventory for Depressive Symptomatology–Clinician-Rated [IDS-C]) [21], early life stress (Childhood Trauma Questionnaire [CTQ]) [22], GAF [10] at baseline and in the past year, Functioning Assessment Short Test (FAST) [23,24], prodromal psychotic symptoms (Prodromal Questionnaire-16 [PQ-16]) [25], bipolar risk factors (EPIbipolar) [18], extended Bipolar-at-Risk criteria (BARS) [26], and Bipolar Prodrome Symptom Scale-Prospective (BPSS-P) [27]. In total, 126 items were included as features (for details, refer to Table S1 in Multimedia Appendix 1). All features were included at the item level, except for PQ-16, which was only available as a total scale score in the study database.

Although more differentiated measures of functional outcome do exist, we used GAF, as it is highly established and largely present in longitudinal datasets (including Early-BipoLife), which enables larger training and validation samples. Rather than predicting GAF as a continuous variable, we dichotomized GAF into impaired and nonimpaired for the following reasons: (1) to include trajectories that remained stable on a low functioning level, as well as those that impaired during the follow-ups, (2) include both follow-ups in one outcome variable, and (3) reflect a hypothetical clinical scenario, where typically a binary decision should be made between assigning and not assigning to an intervention. We defined the negative outcome as GAF equal to or below 60 (ie, moderate symptoms such as flat affect and circumstantial speech, occasional panic attacks, or moderate difficulty in social, occupational, or school functioning, such as few friends, conflicts with peers or coworkers) at least 1 follow-up. This value is in alignment with previously used cutoffs in the psychosis risk literature (Koutsouleris et al [12]: Social and Global Functioning: Role scales ≤7; Lalousis et al [28]: Global Assessment of Functioning-Symptom [GAF-S] score of ≤60). Clinically, functional impairment at this level may be considered as an indication for clinical (outpatient or inpatient) intervention, as compared to GAF 70‐61, which denotes good functioning with only mild symptoms and difficulties.

In post hoc analyses, we aimed to predict the following additional outcomes: (1) higher and lower GAF cutoff values (ie, GAF ≤55 and ≤65), (2) we used time series k-means clustering implemented in Scikit-learn v. 1.5.2 [29] to cluster the GAF trajectories in 2 clusters using 6 time points (GAF values at past year to baseline, baseline, past year to 1-year follow-up, 1-year follow-up, past year to 2-year follow-up, and 2-year follow-up) and differentiate between participants belonging to these clusters. (3) Maximum GAF values over the past year at follow-up 1 or 2, aiming to capture long-term impairments that may have been overlooked by the GAF value at follow-up assessments.

### MRI Acquisition, Preprocessing, and Quality Assessment

We acquired 1 mm isotropic resolution structural T1-weighted images using Siemens Magnetom MR scanners at 6 sites (Trio, Skyra, and Prisma models) and a Philips Achieva scanner at 1 site. We standardized the pulse sequence parameters across all sites to the extent permitted by each platform. For a detailed description of the scanning protocol, including the details of MRI scanners, specific hardware configurations, and pulse sequence parameters, see Vogelbacher et al [30].

Prior to preprocessing, we performed the data acquisition and quality assessment using the MRIQC tool [31]. Two authors visually inspected the obtained reports of several metrics, including a movement plot and a plot of the background noise. In this way, 23 participants were excluded from further analysis due to strong movement (n=18), ghosting (n=1), or fold-over artifacts (n=4).

We preprocessed the T1-weighted structural MRI using Freesurfer 6.0 (Martinos Center for Biomedical Imaging; cortical and subcortical parcellations) and 7.1.1/7.2.0 (Martinos Center for Biomedical Imaging; hippocampal subfields and amygdala nuclei), partially running on the Center for Information Services and High-Performance Computing (ZIH) by TU Dresden [32]. We obtained regional cortical thicknesses and surface area values for 68 cortical brain areas defined by the Desikan-Killiany atlas [33], 14 volumes of subcortical structures [34], 18 amygdala nuclei, 24 volumes of hippocampal subfields [35,36], that is, 124 MRI features in total (refer to Section 2 in Multimedia Appendix 1 for additional details). We performed a standardized quality control of the cortical and subcortical segmentations according to the established protocols of the Enhancing NeuroImaging Genetics through Meta-Analysis (ENIGMA) working group [37]. This included a visual inspection of the segmented regions using the internal and external surface methods, as well as statistical outlier detection. The outliers were subjected to further visual inspection. For details on preprocessing and exclusion of participants who did not pass the quality control or displayed major segmentation errors (n=8), please see our previous publications [38,39].

### Machine Learning Pipelines

First, we predicted the impaired status at follow-up using a linear support vector machine (SVM; LIBSVM 3.1.2; developed by Chih-Chung Chang and Chih-Jen Lin [40], C-SVC, L1-loss) with instance weighting, implemented in the Neurominer Toolbox v. 1.2 (Nikolaos Koutsouleris) [41] using baseline clinical features in 357 participants after excluding those with missing GAF values at any follow-up and further removing participants with missing values for 25% or more of the features.

Second, we trained separate SVM classifiers using baseline clinical and MRI features in 124 participants with available baseline MRI. We then applied stacked generalization, where the decision scores from both base models were combined using another linear SVM that used the decision scores from the completed analyses as input features to predict the outcome group [12].

All pipelines included feature-wise scaling (range 0 to 1) and imputation of missing values using the median of the 7 nearest neighbors (Hamming distance for nominal features with 2 or fewer unique values, Euclidean distance for ordinal features). Covariates sex, age, and intracranial volume were regressed out using partial correlations exclusively in the MRI feature set, as sex and age were considered informative in the clinical domain. Both the removal of nuisance covariates and the imputation of missing data were carried out separately for test and training sets. The imputed values were derived from the training test and applied to the test set.

We used a nested CV scheme, with an outer loop (CV2) using 6 folds (leave-one-site-out, where each site represented a study site) and an inner loop (CV1) using 5 folds for model selection and C parameter tuning. In each inner fold, we applied a wrapper-based greedy feature selection approach to retain the ratio of selected features to total participants at 1:5. As a result, k=71 features were selected for the clinical models and k=25 for the MRI and stacking models. The regularization parameter (C) was optimized across 9 values ranging from 0.00390625 to 256. In the SVM analyses, probability estimation was not enabled. This means the classifier did not use a probabilistic cutoff (such as 0.5) but relied on the standard SVM decision function with a fixed decision boundary.

We assessed the contribution of features toward the classification using the sign-based consistency, which was defined as the consistency of the weights assigned by the model to a given feature, reduced by the fraction of models that removed that feature during the wrapper-based feature selection. Sign-based consistency of 1 means that the weights of the feature all share the same sign and the feature has been selected by all classifiers in the inner CV loop, whereas a value of 0 means that positive and negative weights occurred equally or the given feature was omitted in all CV folds [42].

In order to improve clinical interpretability, we analyzed the most predictive set of features retrieved by the SVM analysis (defined as sign-based consistency score *P*<.05) using a decision tree classifier implemented in Scikit-learn (v. 1.5.2). Briefly, the decision tree minimizes the entropy by splitting the data so that each subset contains as few mixed class labels as possible (ie, each leaf ideally contains a single class). For a binary classification, entropy=1 for a leaf composed of 50% of class 1% and 50% of class 2, whereas entropy =0 for a leaf containing only a single class. Using a 5-fold CV, we trained and evaluated the model using the following parameters: decision criterion=entropy, maximal depth=3 layers (for interpretability reasons, we decided against more layers), minimal sample splits 2‐10, class weighting to account for imbalanced classes. Class predictions were based on the default scikit-learn behavior, in which each leaf node assigns the class with the highest empirical probability (equivalent to an implicit 0.5 threshold in binary classification).

To assess clinical utility, we performed decision curve analysis, calculating net benefit across threshold probabilities and comparing each model with treat-all and treat-none strategies [43].

### LLM Analysis

We used a locally hosted Llama-3 (llama-3.3-70b-instruct-q4km) via the llama.cpp framework on a local hospital computer [6]. For each participant, we converted the clinical assessments into reverse-engineered text notes by substituting item’s scores by their exact descriptions from the assessment instruments (eg, if the participant scored 0 in the item falling asleep of the IDS-C, the text note included the sentence: “I never take longer than 30 min to fall asleep.” Refer to Section 3A in Multimedia Appendix 1 for an example). This process was performed automatically using a Python script. We performed no further processing of text notes. All predictions using the Llama-3.3-70B-Instruct-q4km model were generated using deterministic decoding parameters to ensure reproducibility. We set the temperature to 0, top_p to 1.0, and max_tokens to 256. A fixed random seed was applied for all inference calls. Under these settings, model outputs were identical across repeated runs. The following items were included: age, gender, diagnoses, substance use, IDS-C, CTQ, GAF baseline and past year, FAST, information on past and present psychiatric treatment, and first-degree relatives for bipolar disorder. Llama-3 was instructed using a prompt to classify the functional outcome group (Section 3B in Multimedia Appendix 1). To further investigate the generalizability of the prompting approach, we repeated the analysis in an external sample of 590 participants aged 15‐35 years from the FOR2107 longitudinal sample of persons with affective disorder [44] (refer to Table S2 in Multimedia Appendix 1 for the details of the validation sample). The reverse-engineered notes included age, gender, main diagnosis, HAMD (Hamilton Depression Rating Scale), CTQ items, and GAF baseline.

### Ethical Considerations

The authors assert that all procedures contributing to this work comply with the ethical standards of the relevant national and institutional committees on human experimentation and with the Helsinki Declaration of 1975, as revised in 2013. All procedures involving human subjects or patients were approved by the Ethics Committee of the Medical Faculty at the Technical University Dresden (no. EK290082014) and all local ethics committees. We obtained written informed consent after providing comprehensive information about the study aims and procedures.

## Results

### Demographics

**Figure 1.**
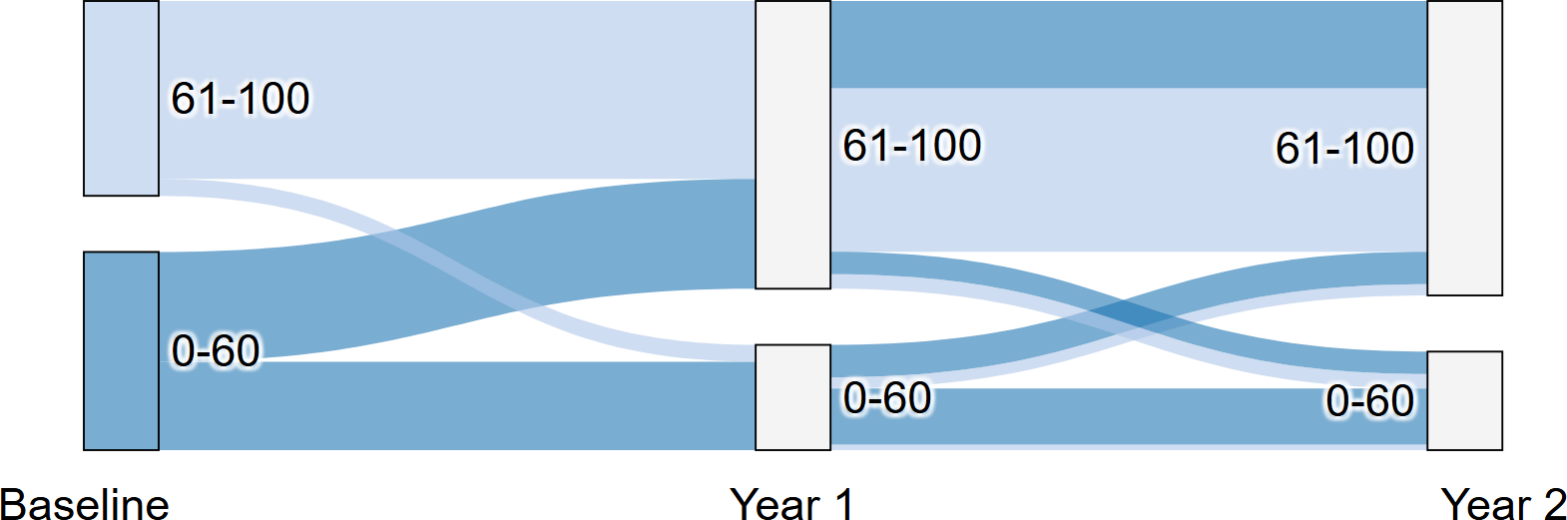
Graphical representation of functional outcome trajectories (the nodes of the Sankey plot represent the Global Assessment of Functioning values [lower value represents higher impairment]).

Overall, the global functioning improved within the 2-year follow-up (mean_baseline_ 61.6, SD 15.7; mean_year1_ 69.2, SD 14.3; mean_year2_ 70.6, SD 14.4). Refer to Figure 1 for the trajectories.

The participants impaired at follow-up fulfilled significantly more frequently criteria for psychiatric diagnoses, particularly affective, anxiety, and substance use disorders (Table 1). They also more frequently contacted mental health services. The nonimpaired group contained more first-degree relatives of persons with bipolar disorder.

The impairments in global functioning at baseline were more pronounced in the impaired group in all domains and items of the FAST inventory (Table S3 in Multimedia Appendix 1). For distributions of GAF scores at follow-ups, refer to Figure S1 in Multimedia Appendix 1.

Participants not included in the analysis due to missing data or failed quality assessment with available GAF at baseline (n=855) displayed better clinical functioning at baseline (mean GAF_discarded_ 64.77, SD 18.87 vs GAF_included_ 61.58, SD 15.73; *P*=.005). Participants who opted for MRI differed neither in their baseline characteristics nor in the functional impairment outcome compared to those who did not (Table S4 in Multimedia Appendix 1). For the breakdown of demographic characteristics to single sites, refer to Table S5 in Multimedia Appendix 1.

**Table 1.** Sample demographics.

Demographics	Nonimpaired (n=228)	Impaired (n=129)	Chi-square test (*df*) or *t* test (*df*)	Cohen d	*P* values
Sex (female), n (%)	128 (56.1)	81 (62.8)	1.50 (1)^g^	0.13	.22
Age (years), mean (SD)	25.8 (4.4)	25.32 (4.6)	−1.13 (355)^h^	0.1	.35
Positive migration status, n (%)	59 (26.9)	32 (27.4)	0.01 (1)^g^	0.29	.94
Diagnoses, n (%)					
Any disorder (SKID^a^)	140 (61.4)	112 (86.8)	25.64 (1)^g^	0.35	.001^b^
Affective disorder	87 (38.2)	75 (58.1)	13.3 (1)^g^	0.35	<.001^c^
Psychotic disorder	1 (0.4)	1 (0.8)	0.17 (1)^g^	0.04	.68
Substance use disorder	14 (6.1)	18 (14.0)	5.89 (1)^g^	0.26	.015^b^
Anxiety disorder	56 (24.6)	61 (47.3)	18.61 (1)^g^	0.35	<.001^c^
ADHD^d^	57 (25.0)	31 (24.0)	0.04 (1)^g^	0.02	.84
Somatoform disorder	10 (4.4)	10 (7.8)	1.77 (1)^g^	0.14	.18
Global functioning, mean (SD)					
GAF^e^ baseline	66.68 (14.8)	52.6 (13.1)	8.92 (349)^h^	1.01	<.001^c^
GAF maximum past 1 year	76.31 (14.1)	64.09 (15.4)	7.545 (348)^h^	0.83	.15
GAF 1-year follow-up	76.6 (9.7)	55.67 (11.3)	17.68 (355)^h^	1.99	.001^c^
GAF maximum past 1 year to 1-year follow-up	78.22 (10.5)	63.54 (12.3)	11.44 (355)^h^	1.28	<.001^c^
GAF 2-year follow-up	77.93 (10.4)	57.7 (10.8)	17.31 (355)^h^	1.91	<.001^c^
GAF maximum past 1 year to 2-year follow-up	79.43 (10.1)	63.8 (11.3)	13.02 (355)^h^	1.46	<.001^c^
Contact to mental health services present, n (%)	140 (61.4)	98 (76.0)	7.92 (1)^g^	0.3	.005^b^
Contact to mental health services past, n (%)	171 (75.0)	115 (89.1)	9.99 (1)^g^	0.33	.002^b^
First-degree relatives with bipolar disorder, n (%)	32 (14.0)	8 (6.2)	5.081 (1)^g^	0.24	.024^b^
Transition to bipolar disorder, n (%)	4 (1.8)	1 (0.8)	0.572 (1)^g^	0.08	.45
PQ-16^f^, mean (SD)	3.16 (2.9)	4.6 (3.2)	4.311 (353)^h^	0.47	.27

a SKID: Structured Clinical Interview for *DSM-IV *(*Diagnostic and Statistical Manual of Mental Disorders *[Fourth Edition]).

b *P*<.05.

c *P*<.001.

d ADHD: attention-deficit hyperactivity disorder.

e GAF: Global Assessment of Functioning,

f PQ-16: Prodromal Questionnaire-16.

g chi-square test value.

h *t* test value.

### SVM Classification

The linear SVM classifier trained on clinical measures alone (sample size, n=357) to predict the impaired status within the 2-year follow-up achieved balanced accuracy 69.2%, sensitivity 64.3%, and specificity 71.9% (to assess classifier performance across the full range of thresholds, see the receiver operating characteristic curve in Figure S2 in Multimedia Appendix 1). Training the classifier using MRI features (sample size n=124) exclusively achieved balanced accuracy 54%, sensitivity 43.5%, and specificity 60.3%. In the same subsample, using only clinical features achieved a balanced accuracy of 73% (sensitivity 65.2% and specificity 80.8%). Combining the clinical and the MRI classifiers via stacking did not improve the performance (balanced accuracy 65.6%, sensitivity 54.3%, and specificity 76.9%).

**Figure 2.**
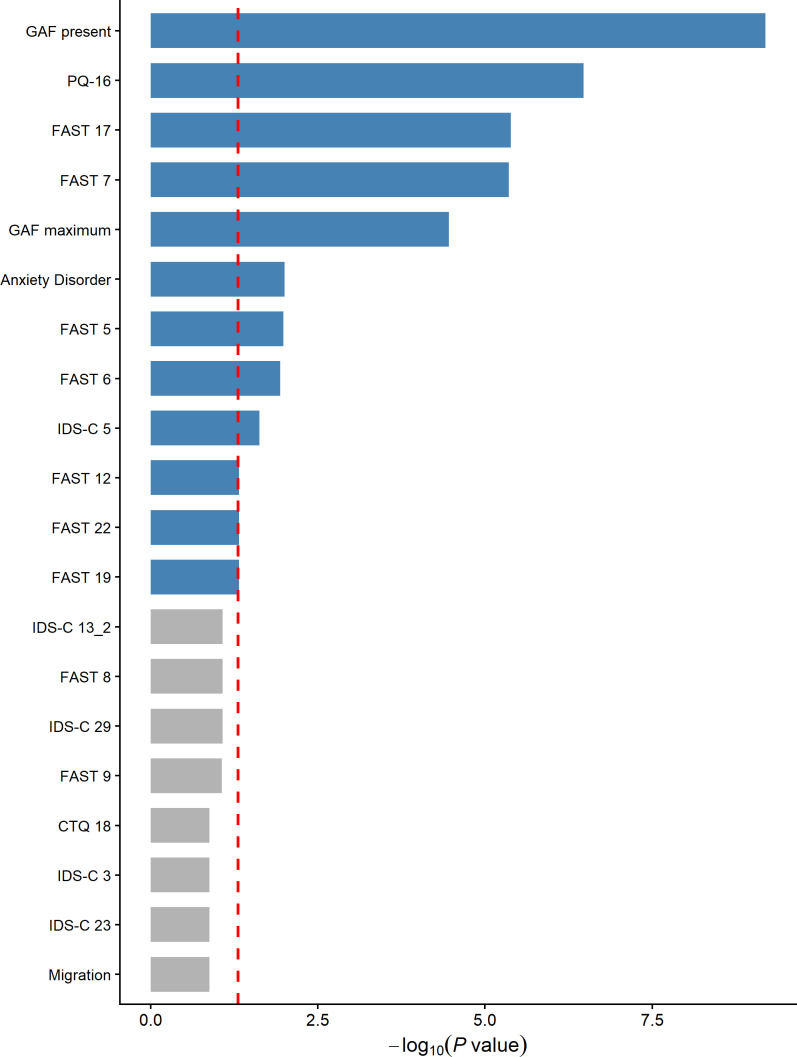
The ranking of clinical features as defined by the sign-based consistency (the red line indicates the significance level *P*<.05). FAST: Functioning Assessment Short Test; GAF: Global Assessment of Functioning; IDS-C: Inventory for Depressive Symptomatology–Clinician-Rated; PQ-16: Prodromal Questionnaire-16.

The most consistently predictive features were items related to occupational functioning, interpersonal relationships, cognitive functioning, psychotic and affective symptoms, as well as the presence of anxiety disorder (Table 2; Figure 2; and Figure S3 in Multimedia Appendix 1). As GAF baseline was retrieved as the most predictive item, we trained an SVM classifier exclusively using GAF baseline as a single feature, achieving a comparable performance (balanced accuracy 70.2%, sensitivity 74.8%, and specificity 65.6%). However, for clinical decision-making, reliable probability estimates are essential, and single-feature classifiers typically yield poor calibration. To evaluate both the accuracy and reliability of probability estimates, we constructed calibration curves for single-feature and multifeature models. The multifeature model achieved a lower Brier score (0.214 vs 0.311), indicating more accurate and reliable probability estimates (Section 4 in Multimedia Appendix 1).

**Table 2.** The list of significant predictive clinical features as defined by the sign-based consistency (see also Figure S3 in Multimedia Appendix 1).

Ranking	Item	Note
1.	GAF^a^ present	Baseline clinical functioning
2.	PQ-16^b^	≥6 positive screening for psychosis
3.	FAST^c^ 17	Interpersonal relationships: maintaining a friendship or friendships
4.	FAST 7	Occupational functioning: working in the field in which you were educated
5.	GAF maximum	Maximum GAF value over the past year prior to baseline
6.	Anxiety disorder	*DSM-IV^d^* diagnosis (SKID-I)^e^
7.	FAST 5	Occupational functioning: holding down a paid job
8.	FAST 6	Occupational functioning: accomplishing tasks as quickly as necessary
9.	IDS-C^f^ 5	Mood (sad)
10.	FAST 12	Cognitive functioning: ability to solve a problem adequately
11.	FAST 19	Interpersonal relationships: having good relationships with people close to you
12.	FAST 22	Interpersonal relationships: being able to defend your interests

a GAF: Global Assessment of Functioning.

b PQ-16: Prodromal Questionnaire 16.

c FAST: Functioning Assessment Short Test.

d *DSM-IV*: *Diagnostic and Statistical Manual of Mental Disorders* (Fourth Edition).

e SKID-I: Structured Clinical Interview for *DSM-IV* Axis I Disorders.

f IDS-C: Inventory for Depressive Symptomatology–Clinician-Rated.

### Decision Tree

**Figure 3.**
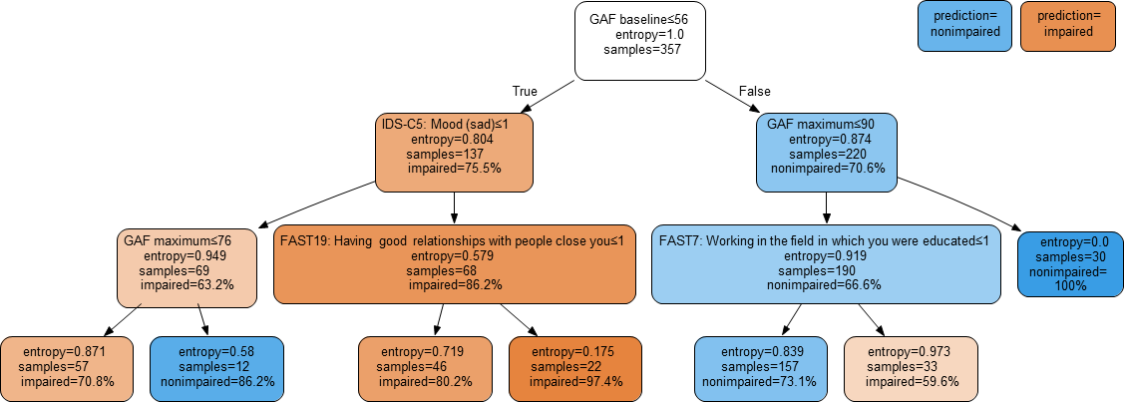
Decision tree predicting the impaired functional outcome within 2-year follow-up. The arrows pointing to the left indicate that the condition is fulfilled (true), the arrows to the right indicate not fulfilled (false). Colors indicate the decision being made by the tree when the condition was fulfilled. The decision tree assigns the leaf label according to the corresponding majority class in that leaf. The number of samples and the percentage of individuals with impaired outcome within each node or leaf are being displayed. FAST: Functioning Assessment Short Test; GAF: Global Assessment of Functioning; IDS-C: Inventory for Depressive Symptomatology–Clinician-Rated.

Training the decision tree using the set of most consistently predictive features (Table 2) retrieved a significant decision tree with the following parameters: balanced accuracy 76.6%, sensitivity 78.3%, and specificity 74.9%. The stratification using baseline GAF was further refined by combining the sequence with the best GAF in the past year and items related to social functioning, early life adversity, and psychosis screening. For the visualization of the decision tree, refer to Figure 3. Training the decision tree using GAF baseline only achieved balanced accuracy 72.7%, sensitivity 67.4%, and specificity 78%.

### Classification Using LLM

Classification using full-text notes yielded balanced accuracy 72.6%, sensitivity 78.3%, and specificity 66.9%. After excluding GAF baseline value, Llama-3 achieved balanced accuracy 69.7%, sensitivity 88.4%, and specificity 51%. In the external sample of persons with affective disorders (Table S2 in Multimedia Appendix 1), LLM achieved balanced accuracy 61.2%, sensitivity 55.4%, and specificity 70%. After excluding GAF, Llama-3 achieved balanced accuracy 60.8%, sensitivity 46.9%, and specificity 74.7%.

### Post Hoc Analyses

Decreasing GAF thresholds for impaired outcome (ie, increasing the impairment) revealed improved balanced accuracies for MRI data after reducing the feature set to hippocampus and amygdala subregions (Table S4 in Multimedia Appendix 1). However, stacking amygdala and hippocampus features with clinical data revealed no improvement (73.0% vs 72.3%, respectively). Data-driven clustering of GAF trajectories using 6 GAF time points into 2 clusters revealed a low- and high-functioning cluster (Figure S4 in Multimedia Appendix 1). However, MRI data were not able to differentiate between the 2 clusters (Table 3). The decision curve analysis for the SVM, decision tree, and LLM showed that neither the decision tree nor the SVM or LLM provided a positive net benefit compared with a “treat none” strategy (Figure S5 in Multimedia Appendix 1). In order to elucidate the relationships between the most predictive variables and functional outcome, we performed partial dependence plot analyses (Figure S6 in Multimedia Appendix 1).

**Table 3.** Balanced accuracies of the support vector machine classifiers for different Global Assessment of Functioning cutoff values as outcomes. The different balanced accuracy values for clinical features and GAF 60 in the main analysis are due to reduced sample size (n=124, ie, participants with magnetic resonance imaging).

GAF^a^	Clinical (%)	sMRI^b^ (%)	Cortical thickness (%)	Hippocampus and amygdala subregions (%)	Stacked clinical+sMRI (%)
GAF 55	78.0	56.5	52.7	58.6	69.4
GAF 60	73.0	51.9	45.4	57.1	65.6
GAF 65	58.2	46.5	47.2	52.2	51.3
GAF clustered	66.6	50.1	52.4	50.1	64.8

a GAF: Global Assessment of Functioning.

b sMRI: structural magnetic resonance imaging.

SVM regression to predict the maximum GAF values over the past year to follow up using clinical features achieved a significant positive relationship between predicted and actual values (*R*²=0.12; *P*<.001) with a mean absolute error (MAE)=10.0 GAF points for clinical data. MRI features revealed a weak relationship (*R*²=0.03; *P*<.04, MAE=11.3) and there was no relevant improvement using stacking (*R*²=0.14; *P*<.001, MAE=9.8). Repeating the primary SVM analysis to predict impaired outcome using different machine learning methods did not improve the balanced accuracies for clinical and structural MRI features (Table S6 in Multimedia Appendix 1).

## Discussion

Machine learning predicted global functioning within the 2-year follow-up with good performance. A detailed analysis of the most predictive features revealed baseline occupational functioning, interpersonal relationships, cognitive functioning, psychotic, affective, as well as anxiety symptoms as most predictive. The feature set was further refined using a decision tree, providing an interpretable solution for possible clinical translation. Native, “out-of-the-box” LLM achieved comparable performance using clinical data. Although MRI did not improve the prediction achieved by features based on clinical assessments only, amygdala and hippocampal subregions were more predictive than other MRI features, especially in individuals with lower functioning at follow-up.

SVM predicted the functional outcome with good accuracy using baseline clinical data. This is in alignment with [12,12], where machine learning predicted the 1-year functioning in individuals with clinical high risk for psychosis, although the accuracy in our sample was lower (69.2% vs 76.9%). This was most likely due to different populations between the cohorts, the latter containing mostly participants with affective and anxiety symptoms and only a minority with psychotic symptoms (average PQ-16 was below 6, which denotes negative psychosis prodrome screening; less than 1% fulfilled the *DSM-IV* criteria for psychotic disorder). Individuals with predominantly affective and anxiety symptoms might display more heterogeneous baseline symptoms as well as trajectories than persons with clinical high risk for psychosis. This might lead to less accurate predictive models.

In contrast to the clinical high risk for psychosis sample [12], structural MRI in our sample did not improve the prediction beyond what was already achieved using clinical data. In models using structural neuroimaging only, Koutsouleris et al [12] achieved balanced accuracy up to 76.2%. Whereas patients with schizophrenia display the most prominent structural alterations among major psychiatric disorders (excluding dementia), persons with affective anxiety disorders and ADHD tend to display less pronounced structural alterations than persons with psychosis [45]. The concept of clinical high risk for psychosis encompasses persons with global structural deficits [46]. Interestingly, reducing the set of features to hippocampus and amygdala subregions improved the prediction accuracy of MRI data in our sample, especially at lower thresholds of GAF, that is, while detecting individuals with poorer functional outcome. Indeed, hippocampus and amygdala tend to display structural alterations in affective disorders [47,48]. Focusing on these regions, even using multimodal or high-resolution MRI data [49], might further improve predictions. On the other hand, as their incremental predictive value beyond clinical measures was modest, our study does not support routine MRI scanning given the considerable cost, limited availability, and additional patient burden.

Specificity exceeded sensitivity across all models. Yet, in early-intervention settings, maximizing sensitivity is often more clinically meaningful, as the priority is to identify individuals at risk. For implementation, sensitivity could be increased by modifying the decision threshold of probabilistic models (eg, lowering the SVM probability cutoff), allowing the model to favor true-positive detection. A data-driven selection of the most predictive variables revealed a limited set of features related to occupational functioning, interpersonal relationships, cognitive functioning, psychotic, affective, as well as anxiety symptoms as most predictive. For the SVM classifier, baseline GAF emerged as the single most predictive feature, and adding further clinical items did not increase overall classification accuracy. However, accuracy alone does not capture the quality of the probability estimates produced by the model. In clinical decision-making, well-calibrated probabilities are often more important than discrete class labels, as they allow clinicians to gauge the degree of risk and tailor interventions accordingly. Although baseline GAF largely drives classification performance, incorporating additional features produces smoother and more reliable probability estimates across individuals (refer to Section 4 in Multimedia Appendix 1). In this regard, the multifeature SVM outperformed the single-feature model, highlighting that models with richer input information can provide better-calibrated confidence estimates even when a single predictor dominates overall accuracy. This improved calibration may enhance clinical utility by supporting risk-stratified decision-making rather than binary classification. In contrast, the inclusion of additional features led to a modest improvement in performance for the decision tree classifier.

Interestingly, Llama-3 predicted functional outcome with a balanced accuracy comparable to trained SVM or decision tree classifiers. While it did not outperform these traditional models, it is important to emphasize that we used a native, “out-of-the-box” general-purpose LLM, not primarily trained for predicting clinical outcomes. Prompt-engineering [50] or fine-tuning might further improve predictive performance [7]. A significant disadvantage of LLMs is the lack of interpretability, bias risk due to nondisclosed training data [51] as well as of reliable probability estimates [52]. These issues need to be addressed in order to bring LLMs to the bedside. In order to explore the potential of LLMs for clinical predictions, unstructured text, such as clinical notes or interview transcripts, should become an integral part of longitudinal studies.

In this study, we mapped the trajectories of global functioning using GAF at follow-up as well as maximum GAF over the past year (assessed retrospectively). Although the GAF provides useful historical continuity and comparability with prior BipoLife and FOR2107 longitudinal studies, it has known limitations as it partly conflates symptom severity with functioning. We used the GAF to maintain consistency with the legacy datasets and to allow meaningful longitudinal comparisons across baseline and follow-up. Nonetheless, functioning-specific measures such as the SOFAS (Social and Occupational Functioning Assessment Scale) [53], which avoid symptom contamination and are increasingly recommended for youth and transdiagnostic settings, may provide greater construct validity for future predictive modeling. Other more suitable measures for tracking long-term global functioning include “days out of role” (ie, the number of days an individual is unable to work or perform daily activities due to physical or mental health issues) [54] and “time use” (ie, weekly engagement in structured activities) [55]. Future longitudinal studies should incorporate more differentiated long-term assessments.

Interestingly, the number of first-degree relatives of bipolar patients, as well as transitions to bipolar disorder, was higher in the nonimpaired group. This might suggest that persons with manic episodes might have better functioning in the long run, at least in the early stages. However, the number of transitions was too low to be statistically significant. Moreover, some individuals in the sample with depressive symptoms might have unrecognized bipolar disorder that would be revealed with a longer follow-up period.

We used a conservative leave-one-site-out validation without data harmonization. Although harmonization might improve the performance of structural MRI-based models, it introduces a problem of data leakage between train and test sets. An external validation might provide better insights in generalizability of predictive models; however, as none of the models achieved >80% accuracy and therefore relevance for clinical use [56,57], the prediction models require further refinement prior to external validation. Multimodal data such as genetics, immunological markers [28], clinician prognostic estimates, or natural language processing features [6,58] could improve classification. In addition, functional outcome measures should map functional trajectories with sufficient temporal resolution.

In a transdiagnostic cohort of young individuals, machine learning and LLM can predict 2-year global functioning with good performance. Key predictors included occupational functioning, interpersonal relationships, cognitive functioning, psychotic and affective symptoms, as well as anxiety symptoms. Although hippocampal and amygdala subregions showed potential as more predictive MRI features, particularly in those with poorer outcomes, MRI data did not enhance overall performance. Decision curve analysis revealed that none of the 3 models provided net benefit beyond a “treat-none” strategy, suggesting limited clinical advantage of model-based decision-making in this cohort. Further refinements of predictors as well as functional outcome measures are needed to better reflect clinical trajectories and enhance predictive accuracy.

## Supplementary material

10.2196/84424 Multimedia Appendix 1 Supplementary information.

## Abbreviations

ADHD: attention-deficit hyperactivity disorder
BARS: extended Bipolar-at-Risk criteria
BPSS-P: Bipolar Prodrome Symptom Scale-Prospective
CTQ: Childhood Trauma Questionnaire
CV: cross-validation
*DSM-IV*: *Diagnostic and Statistical Manual of Mental Disorders* (Fourth Edition)
ENIGMA: Enhancing NeuroImaging Genetics through Meta-Analysis
FAST: Functioning Assessment Short Test
GAF: Global Assessment of Functioning
GAF-S: Global Assessment of Functioning-Symptom
HAMD: Hamilton Depression Rating Scale
IDS-C: Inventory for Depressive Symptomatology–Clinician-Rated
LLM: large language model
MAE: mean absolute error
MRI: magnetic resonance imaging
PQ-16: Prodromal Questionnaire-16
SKID-I: Structured Clinical Interview for *DSM-IV* Axis I Disorders
SOFAS: Social and Occupational Functioning Assessment Scale
SVM: support vector machine
ZIH: Center for Information Services and High-Performance Computing

## References

[1] (2024). Pension insurance in time series. German Pension Insurance.

[2] Arias D, Saxena S, Verguet S (2022). Quantifying the global burden of mental disorders and their economic value. EClinicalMedicine.

[3] Kjell ONE, Kjell K, Schwartz HA (2024). Beyond rating scales: With targeted evaluation, large language models are poised for psychological assessment. Psychiatry Res.

[4] Goh E, Gallo R, Hom J (2024). Large language model influence on diagnostic reasoning: a randomized clinical trial. JAMA Netw Open.

[5] Buckley TA, Crowe B, Abdulnour REE, Rodman A, Manrai AK (2025). Comparison of frontier open-source and proprietary large language models for complex diagnoses. JAMA Health Forum.

[6] Wiest IC, Verhees FG, Ferber D (2024). Detection of suicidality from medical text using privacy-preserving large language models. Br J Psychiatry.

[7] Ben Shoham O, Rappoport N (2024). CPLLM: clinical prediction with large language models. PLOS Digit Health.

[8] Brown KE, Yan C, Li Z (2025). Large language models are less effective at clinical prediction tasks than locally trained machine learning models. J Am Med Inform Assoc.

[9] Yang Z, Mitra A, Liu W, Berlowitz D, Yu H (2023). TransformEHR: transformer-based encoder-decoder generative model to enhance prediction of disease outcomes using electronic health records. Nat Commun.

[10] Aas IHM (2011). Guidelines for rating Global Assessment of Functioning (GAF). Ann Gen Psychiatry.

[11] Cowman M, Godfrey E, Walsh T (2024). Measures of social and occupational function in early psychosis: a systematic review and meta-analysis. Schizophr Bull.

[12] Koutsouleris N, Kambeitz-Ilankovic L, Ruhrmann S (2018). Prediction models of functional outcomes for individuals in the clinical high-risk state for psychosis or with recent-onset depression: a multimodal, multisite machine learning analysis. JAMA Psychiatry.

[13] Slot MIE, Urquijo Castro MF, Winter-van Rossum I (2024). Multivariable prediction of functional outcome after first-episode psychosis: a crossover validation approach in EUFEST and PSYSCAN. Schizophrenia (Heidelb).

[14] de Nijs J, Burger TJ, Janssen RJ (2021). Individualized prediction of three- and six-year outcomes of psychosis in a longitudinal multicenter study: a machine learning approach. NPJ Schizophr.

[15] Oomen PP, Begemann MJH, Brand BA (2023). Longitudinal clinical and functional outcome in distinct cognitive subgroups of first-episode psychosis: a cluster analysis. Psychol Med.

[16] Arribas M, Oliver D, Patel R (2024). A transdiagnostic prodrome for severe mental disorders: an electronic health record study. Mol Psychiatry.

[17] Pfennig A, Leopold K, Martini J (2020). Improving early recognition and intervention in people at increased risk for the development of bipolar disorder: study protocol of a prospective-longitudinal, naturalistic cohort study (Early-BipoLife). Int J Bipolar Disord.

[18] Leopold K, Ritter P, Correll CU (2012). Risk constellations prior to the development of bipolar disorders: rationale of a new risk assessment tool. J Affect Disord.

[19] Martini J, Bröckel KL, Leopold K (2024). Young people at risk for developing bipolar disorder: Two-year findings from the multicenter prospective, naturalistic Early-BipoLife study. Eur Neuropsychopharmacol.

[20] Wittchen HU, Zaudig M, Fydrich T, SKID (1997). Achse I Und II: Achse I: Psychische Störungen; Achse II: Persönlichkeitstörungen 1 Auflage.

[21] Rush AJ, Giles DE, Schlesser MA, Fulton CL, Weissenburger J, Burns C (1986). The Inventory for Depressive Symptomatology (IDS): preliminary findings. Psychiatry Res.

[22] Bernstein DP, Fink L, Handelsman L, Foote J (2011). Childhood trauma questionnaire. APA PsycNet.

[23] Rosa AR, Sánchez-Moreno J, Martínez-Aran A (2007). Validity and reliability of the Functioning Assessment Short Test (FAST) in bipolar disorder. Clin Pract Epidemiol Ment Health.

[24] Christensen MC, Schmidt SN, Grande I (2024). The Functioning Assessment Short Test (FAST): clinically meaningful response threshold in patients with major depressive disorder receiving antidepressant treatment. J Affect Disord.

[25] Ising HK, Veling W, Loewy RL (2012). The validity of the 16-item version of the Prodromal Questionnaire (PQ-16) to screen for ultra high risk of developing psychosis in the general help-seeking population. Schizophr Bull.

[26] Bechdolf A, Ratheesh A, Cotton SM (2014). The predictive validity of bipolar at-risk (prodromal) criteria in help-seeking adolescents and young adults: a prospective study. Bipolar Disord.

[27] Correll CU, Olvet DM, Auther AM (2014). The Bipolar Prodrome Symptom Interview and Scale-Prospective (BPSS-P): description and validation in a psychiatric sample and healthy controls. Bipolar Disord.

[28] Lalousis PA, Schmaal L, Wood SJ (2022). Neurobiologically based stratification of recent-onset depression and psychosis: identification of two distinct transdiagnostic phenotypes. Biol Psychiatry.

[29] Scikit-learn: machine learning in python. scikit-learn.

[30] Vogelbacher C, Möbius TWD, Sommer J (2018). The Marburg-Münster Affective Disorders Cohort Study (MACS): a quality assurance protocol for MR neuroimaging data. Neuroimage.

[31] Esteban O, Birman D, Schaer M, Koyejo OO, Poldrack RA, Gorgolewski KJ, Bernhardt BC (2017). MRIQC: advancing the automatic prediction of image quality in MRI from unseen sites. PLoS One.

[32] Data intensive computing – high performance computing. Dresden University of Technology.

[33] Desikan RS, Ségonne F, Fischl B (2006). An automated labeling system for subdividing the human cerebral cortex on MRI scans into gyral based regions of interest. Neuroimage.

[34] Fischl B, van der Kouwe A, Destrieux C (2004). Automatically parcellating the human cerebral cortex. Cereb Cortex.

[35] Iglesias JE, Augustinack JC, Nguyen K (2015). A computational atlas of the hippocampal formation using ex vivo, ultra-high resolution MRI: application to adaptive segmentation of in vivo MRI. Neuroimage.

[36] Iglesias JE, Van Leemput K, Augustinack J (2016). Bayesian longitudinal segmentation of hippocampal substructures in brain MRI using subject-specific atlases. Neuroimage.

[37] Structural image processing protocols. ENIGMA.

[38] Mikolas P, Marxen M, Riedel P (2024). Prediction of estimated risk for bipolar disorder using machine learning and structural MRI features. Psychol Med.

[39] Huth F, Tozzi L, Marxen M (2023). Machine learning prediction of estimated risk for bipolar disorders using hippocampal subfield and amygdala nuclei volumes. Brain Sci.

[40] LIBSVM: a library for support vector machines. ACM Digital Library.

[41] Neurominer-git/neurominer_1.2. GitHub.

[42] Koutsouleris N, Dwyer DB, Degenhardt F (2021). Multimodal machine learning workflows for prediction of psychosis in patients with clinical high-risk syndromes and recent-onset depression. JAMA Psychiatry.

[43] Fitzgerald M, Saville BR, Lewis RJ (2015). Decision curve analysis. JAMA.

[44] Kircher T, Wöhr M, Nenadic I (2019). Neurobiology of the major psychoses: a translational perspective on brain structure and function-the FOR2107 consortium. Eur Arch Psychiatry Clin Neurosci.

[45] Ching CRK, Hibar DP, Gurholt TP (2022). What we learn about bipolar disorder from large-scale neuroimaging: findings and future directions from the ENIGMA Bipolar Disorder Working Group. Hum Brain Mapp.

[46] Vissink CE, Winter-van Rossum I, Cannon TD, Fusar-Poli P, Kahn RS, Bossong MG (2022). Structural brain volumes of individuals at clinical high risk for psychosis: a meta-analysis. Biol Psychiatry Glob Open Sci.

[47] Schmaal L, Hibar DP, Sämann PG (2017). Cortical abnormalities in adults and adolescents with major depression based on brain scans from 20 cohorts worldwide in the ENIGMA Major Depressive Disorder Working Group. Mol Psychiatry.

[48] Hibar DP, Westlye LT, van Erp TGM (2016). Subcortical volumetric abnormalities in bipolar disorder. Mol Psychiatry.

[49] Ebneabbasi A, Mahdipour M, Nejati V (2021). Emotion processing and regulation in major depressive disorder: a 7T resting-state fMRI study. Hum Brain Mapp.

[50] Verhees FG, Huth F, Meyer V (2025). clickBrick Prompt Engineering: Optimizing Large Language Model Performance in Clinical Psychiatry.

[51] Zack T, Lehman E, Suzgun M (2024). Assessing the potential of GPT-4 to perpetuate racial and gender biases in health care: a model evaluation study. Lancet Digit Health.

[52] Gu B, Desai RJ, Lin KJ, Yang J (2024). Probabilistic medical predictions of large language models. NPJ Digit Med.

[53] Rybarczyk B, Kreutzer JS, DeLuca J, Caplan B (2011). Encyclopedia of Clinical Neuropsychology.

[54] Alonso J, Petukhova M, Vilagut G (2011). Days out of role due to common physical and mental conditions: results from the WHO World Mental Health surveys. Mol Psychiatry.

[55] Hodgekins J, French P, Birchwood M (2015). Comparing time use in individuals at different stages of psychosis and a non-clinical comparison group. Schizophr Res.

[56] Nunes A, Schnack HG, Ching CRK (2020). Using structural MRI to identify bipolar disorders - 13 site machine learning study in 3020 individuals from the ENIGMA Bipolar Disorders Working Group. Mol Psychiatry.

[57] Radua J, Carvalho AF (2021). Route map for machine learning in psychiatry: absence of bias, reproducibility, and utility. Eur Neuropsychopharmacol.

[58] Irving J, Patel R, Oliver D (2021). Using natural language processing on electronic health records to enhance detection and prediction of psychosis risk. Schizophr Bull.

[59] Pmiko/bl_gaf. GitHub.

