# Supplementary material for: Functional Outcome Prediction in Young Adults With Mental Health Symptoms Using Machine Learning and Large Language Models: Longitudinal Observational Study
Source: JMIR Ment Health. 2026 Jun 22;13:e84424. doi: 10.2196/84424 (PMC13286077; doi:10.2196/84424)

**Section 1:** Inclusion and exclusion criteria for the three recruitment pathways. For a detailed description of data collection procedures see Pfennig et al. (2020) and Martini et al. (2024). Briefly, we recruited participants in three recruitment pathways: 1) Via consulting early detection centers/facilities and screening positive for ≥1 proposed risk factor for bipolar disorder, 2) young in- and outpatients with a depressive syndrome and patients with an established diagnosis of ADHD.

Inclusion criteria

1. Youth and young adults consulting early recognition centres/facilities:

• Age: 15 to 35 years

• Consultation of an early recognition centre/facility

• Presence of at least one of the proposed risk factors for bipolar disorder: Family history of

bipolar disorder, (sub)threshold affective symptomatology/depressive syndrome,

hypomanic/mood swings, disturbances of circadian rhythm/sleep other clinical hints

2. Young individuals with diagnosed depression:

• Age: 15 to 35 years

• In- or outpatients with a depressive syndrome in the context of: Major depressive disorder,

dysthymic disorder, cyclothymic disorder, minor depressive disorder, recurrent brief

depressive disorder, adjustment disorder with depressed mood, depressive disorder Not

Otherwise Specified (NOS)

3. Patients with ADHD:

• Age: 15 to 35 years

• In- or outpatients with a clinically confirmed ADHD diagnosis

Exclusion criteria:

• Diagnosis of: bipolar disorder, schizoaffective disorder, schizophrenia

• Diagnosis of anxiety, obsessive–compulsive or substance dependence disorder that fully

explains the whole symptomatology

• Limited ability to comprehend the study

• Implied expressed negative declaration of intent to participate in the study by a minor and

• Acute suicidality

**Section 2: Detailed MRI pre-processing information.**

MRI preprocessing followed standard FreeSurfer pipelines. Cortical reconstruction and volumetric segmentation were performed with FreeSurfer 6.0 (recon-all) including skull stripping, intensity normalization, surface extraction, and parcellation according to the Desikan–Killiany atlas. Subcortical segmentation was conducted using aseg/asegstats outputs. Hippocampal subfield and amygdala nuclei segmentations were generated using FreeSurfer versions 7.1.1/7.2.0 (segmentHA_T1.sh).

**Section 3:** A. A hypothetical example of a text note generated from numerical baseline data by substituting item values by their descriptions (abbreviated). B. Llama-3 was instructed using following prompt.

A.

“Sex: Female. Age: 21. Psychotic prodrome (less or equal to 6 screens negative for psychosis): 5. I never take longer than 30 minutes to fall asleep. I do not wake up at night. I feel sad less than half the time. I do not feel irritable. I feel anxious (tense) less than half the time. My mood brightens to a normal level which lasts for several hours when good events occur. I have not had a change in my weight. I have not had a change in my weight. Most of the time, I struggle to focus my attention or to make decisions. I didn't have enough to eat: Never true. I knew there was someone to take care of me and protect me: Very often true. People in my family called me things like stupid, lazy, or ugly: Rarely true. My parents were too drunk or high to take care of the family: Never true. There was someone in my family who helped me feel that I was important or special: Often true. Migration backround: No. Psychotropic_Medication use: No. DSM-IV Affective Disorder: No. DSM-IV Schizoaffective Disorder: No. DSM-IV Substance Use Disorder: No. DSM-IV Anxiety Disorder: Yes. DSM-IV Somatoform Disorder: No. DSM-IV Eating Disorder: No. DSM-IV Adaptive Disorder: No. Psych_Treatment_present: Yes. Psych_Treatment_past: Yes. I had or have first degree relatives with bipolar disorder: No.”

B.

Prompt:

“Given following information, decide, if the person is likely to suffer from impaired functional outcome within the next two years. The impairment is defined as Global Assessment of Functioning (GAF) below 60 points. You have to decide.”.

**Section 4:** Comparison of probability estimations between single- and multiple-feature SVM classifiers.

GAF baseline was retrieved as the most predictive item in the SVM classification. Indeed, a SVM classifier trained exclusively using GAF baseline as a single feature achieved a comparable performance to multi-feature classifier. However, for clinical decision making, probability estimates are more relevant than a categorical response. In order to compare the accuracy and reliability of estimated probabilities, we extracted the estimated probability values from Neurominer Toolbox and constructed calibration curves for both models using the CalibratedClassifierCV function from Scikitlearn. The multi-feature classifier achieved a lower Brier score (0.214 versus 0.311), suggesting a more accurate and reliable probability estimates.


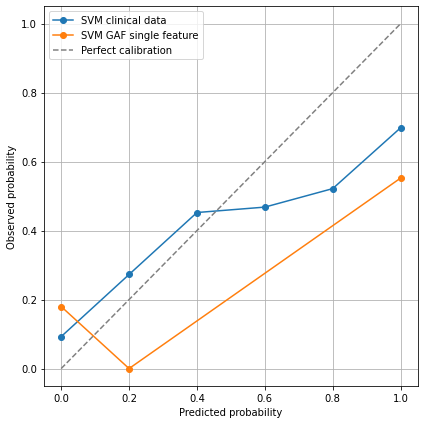


**Table S1.** Clinical assessment instruments.

| **Assessment tool** | **Description** | **Number of items** |
| --- | --- | --- |
| SKID-I | DSM-IV diagnoses (grouped into categories, see Table 1) | 7 |
| Substance use | Smoking, cannabis, amphetamines, opioids, cocaine present (= recent 6 months) / past / never (SKID-I) | 4 |
| Inventory for Depressive Symptomatology - Clinician (IDSC) | Depressive symptoms | 32 |
| Childhood Trauma Questionnaire (CTQ) | Early life stress | 28 |
| Global Assessment of Functioning (GAF) | Global functioning at baseline and in the past year | 2 |
| Functioning Assessment Short Test (FAST) | Impairment or disability in autonomy, occupational functioning, cognitive functioning, financial issues, interpersonal relationships and leisure time | 24 |
| Prodromal Questionnaire (PQ16) | Prodromal psychotic symptoms (total score) | 1 |
| EPIbipolar | Risk symptoms / syndromes for bipolar disorder | 13 |
| Bipolar-at-Risk criteria (BARS) | Risk symptoms / syndromes for bipolar disorder | 5 |
| Bipolar Prodome Symptom Scale (BPSS-P) | Risk symptoms / syndromes for bipolar disorder | 2 |

**Table S2.** The demographics of the external sample of persons with affective disorders drawn from the FOR2107 cohort. This sample was used exclusively for validation of the prompt-based analysis using LLM. Similarly to the primary LLM analysis in the BipoLife sample, we derived synthetic clinical notes based on clinical items (age, gender, diagnoses, HAMD21, CTQ). The impaired outcome was defined as GAF ≤ 60 at the 2-year follow-up. LLM was prompted to derive the categorical decisions based on these notes exclusively.

|  | **Non-impaired** | **Impaired** | **Test** |
| --- | --- | --- | --- |
| N = 590 | 319 | 271 | n/a |
| Sex female (%) | 197 (61.8) | 176 (64.9) | Chi = 0.377, p = 0.539, d = 0.05 |
| Age (SD) | 26.0 (4.6) | 26.4 (4.3) | T = -1.155, p = 0.124, d = 0.09 |
| Diagnoses (%) |  |  | Chi = 35.083, p < 0.001, d = 0.27 |
| MDD | 205 (64.3) | 127 (46.9) |  |
| Anxiety and comorbid MDD | 55 (17.2) | 71 (26.2) |  |
| Bipolar disorder | 27 (8.5) | 23 (8.5) |  |
| Other diagnoses | 32 (10.0) | 49 (18.0) |  |
| Global functioning (SD) |  |  |  |
| GAF baseline | 68.0 (13.6) | 58.9 (12.5) | T = 8.433, p < 0.001, d = 0.7 |
| GAF 2-years follow-up | 76.3 (9.7) | 52.3 (7.8) | T = 24.021, p < 0.001, d = 2.73 |
| HAMD21 | 7.7 (6.4) | 10.7 (10.0) | T = -4.284, p < 0.001, d = 0.36 |
| CTQ sum score | 41.3 (17.9) | 43.7 (19.4) | T = -1.560, p < 0.06, d = 0.13 |

**Table S3.** Baseline clinical functioning according to FAST items between the functionally impaired and non-impaired groups. FAST is a brief, structured instrument specifically designed to evaluate functional impairments in psychiatric populations, with a particular focus on individuals with bipolar disorder. It comprises 24 items that assess impairment or disability in six specific areas of functioning: autonomy, occupational functioning, cognitive functioning, financial issues, interpersonal relationships and leisure time Each item was scored 0 to 3 points.

|  | **Non-impaired** | **Impaired** | **Test** |
| --- | --- | --- | --- |
| Autonomy | | | |
| 1. Taking responsibility for a household | 0.6 (0.87) | 1.07 (1.01) | T = -4.62, p < 0.001, d = 0.5 |
| 2. Living on your own | 0.6 (0.94) | 0.86 (1.04) | T = -2.415, p = 0.016, d = 0.26 |
| 3. Doing the shopping | 0.32 (0.67) | 0.66 (0.9) | T = -4.088, p < 0.001, d = 0.43 |
| 4. Taking care of yourself (physical aspects, hygiene) | 0.14 (0.44) | 0.34 (0.75) | T = -3.158, p = 0.002, d = 0.33 |
| Occupational functioning | | | |
| 5. Holding down a paid job | 0.88 (1.21) | 1.73 (1.33) | T = -6.141, p < 0.001, d = 0.67 |
| 6. Accomplishing tasks as quickly as necessary | 0.92 (1.11) | 1.66 (1.22) | T = -5.805, p < 0.001, d = 0.634 |
| 7. Working in the field in which you were educated | 0.71 (1.14) | 1.56 (1.33) | T = -6.332, p < 0.001, d = 0.686 |
| 8. Occupational earnings | 0.65 (1.12) | 1.26 (1.38) | T = -4.49, p < 0.001, d = 0.49 |
| 9. Managing the expected work load | 0.98 (1.11) | 1.8 (1.2) | T = -6.523, p < 0.001, d = 0.71 |
| Cognitive functioning | | | |
| 10. Ability to concentrate on a book, film | 0.77 (0.88) | 1.26 (0.99) | T = -5.184, p < 0.001, d = 0.52 |
| 11. Ability to make mental calculations | 0.43 (0.74) | 0.67 (0.96) | T = -2.633, p = 0.009, d = 0.28 |
| 12. Ability to solve a problem adequately | 0.38 (0.66) | 0.6 (0.84) | T = - 2.756, p = 0.006, d = 0.29 |
| 13. Ability to remember newly-learned names | 0.64 (0.82) | 1.06 (1.01) | T = -4.216, p < 0.001, d = 0.46 |
| 14. Ability to learn new information | 0.51 (0.74) | 0.8 (0.89) | T = -3.269, p < 0.001, d = 0.35 |
| Financial issues | | | |
| 15. Managing your own money | 0.3 (0.68) | 0.6 (1.0) | T = -3.386, p < 0.001, d = 0.35 |
| 16. Spending money in a balanced way | 0.32 (0.66) | 0.61 (0.91) | T = -3.426, p = 0.002, d = 0.37 |
| Interpersonal relationships |  |  |  |
| 17. Maintaining a friendship or friendships | 0.54 (0.8) | 1.29 (1.04) | T = -7.6, p < 0.001, d = 0.81 |
| 18. Participating in social activities | 0.54 (0.8) | 1.23 (1.12) | T= -6.739, p < 0.001, d = 0.71 |
| 19. Having good relationships with people close you | 0.32 (0.63) | 0.82 (0.99) | T = -5.772, p < 0.001, d = 0.6 |
| 20. Living together with your family | 0.67 (0.9) | 1.09 (1.08) | T = -3.878, p < 0.001, d = 0.42 |
| 21. Having satisfactory sexual relationships | 0.84 (1.08) | 1.23 (1.23) | T = -3.148, p = 0.002, d = 0.34 |
| 22. Being able to defend your interests | 0.64 (0.82) | 1.15 (1.04) | T= -5.112, p < 0.001, d = 0.55 |
| Leisure time | | | |
| 23. Doing exercise or participating in sport | 0.48 (0.76) | 0.93 (1.03) | T= -4.633, p < 0.001, d = 0.5 |
| 24. Having hobbies or personal interests | 0.65 (0.95) | 1.22 (1.06) | T = -5.147, p < 0.001, d = 0.57 |

**Table S4.** Demographic characteristics of participants who opted versus did not opt for MRI.

|  | **MRI** | **Without MRI** | **Test** |
| --- | --- | --- | --- |
| N = 357 | 124 | 233 | n/a |
| Sex female (%) | 71 (57.2) | 138 (59.2) | Chi = 0.129, p = 0.719, d = 0.04 |
| Age (SD) | 25.1 (4.3) | 24.9 (4.6) | T = -0.459, p = 0.646, d = 0.05 |
| GAF baseline | 62.9 (15.1) | 60.9 (16.1) | T = -1.109, p = 0.268, d = 0.13 |
| 2- year functional impairment (GAF ≤ 60) (%) | 46 (37.0) | 83 (35.6) | Chi = 0.076, p = 0.782, d = 0.03 |

**Table S5.** Breakdown of demographics and global functioning per study sites.

|  | **Dresden** | **Frankfurt** | **Berlin (+ Neuruppin)** | **Hamburg** | **Tübingen** | **Marburg** |
| --- | --- | --- | --- | --- | --- | --- |
| N | 71 | 77 | 68 | 43 | 39 | 59 |
| Age (SD) | 25 (4.1) | 25.6 (4.7) | 26.1 (4.8) | 23.4 (5.7) | 24.3 (3.6) | 24.5 (3.4) |
| Sex female (%) | 45 (63.4) | 41 (53.2) | 36 (52.9) | 25 (58.1) | 26 (66.7) | 36 (61.0) |
| GAF baseline (SD) | 59.11 (16.0) | 61.9 (16.1) | 61.2 (14.2) | 56.8 (15.1) | 60.2 (17.8) | 69.4 (13.2) |
| GAF follow-up  impaired (%) | 23 (32.4) | 27 (35.1) | 26 (38.2) | 19 (44.2) | 16 (41.0) | 18 (30.5) |

**Table S6. Prediction of impaired outcome within 2-year follow-up using different ML models.**

|  | **Clinical** | **sMRI** |
| --- | --- | --- |
| Non-linear SVM (RBF) | 65.8% | 55.3% |
| Gradient Boost | 65.4% | 54.3% |
| Random Forest | 68.7% | 48.8% |

**Figure S1.** Distributions of GAF scores at 1-year (A) and 2-year follow up.

A. B.


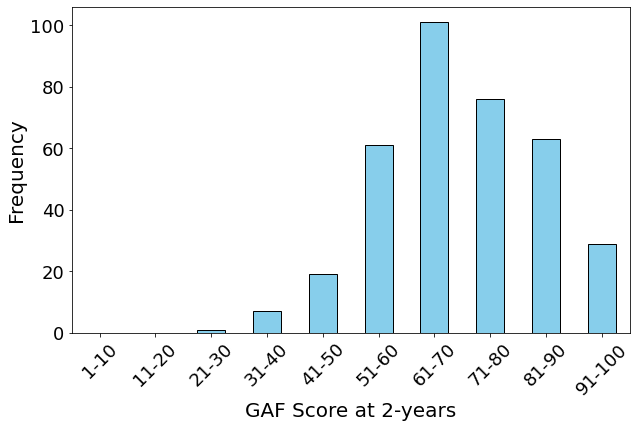

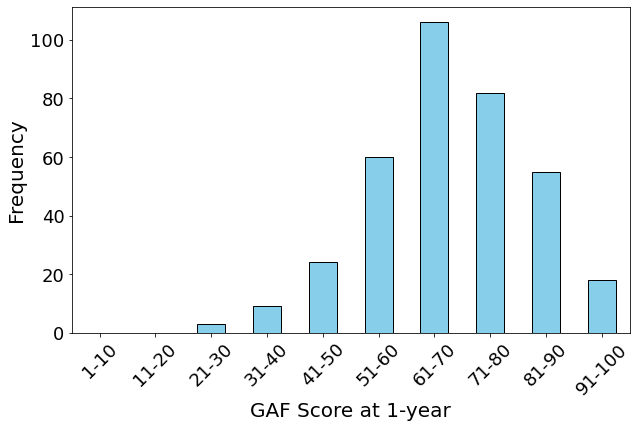


**Figure S2.** The ROC curve for the SVM (A.) and decision tree (B.) classifiers. The ROC curves display the sensitivity – specificity trade-off allowing to assess classifier performance across the full range of thresholds.


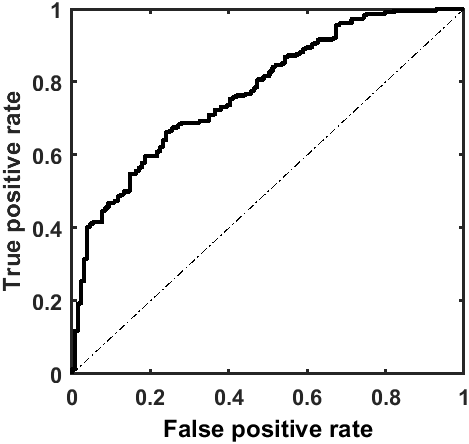
A.


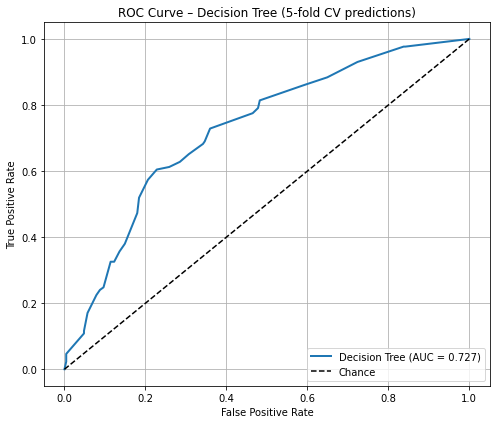
B.

**Figure S3.** Average feature weights of the significantly contributing features. Positive feature weights indicate relative increases, whereas negative weights decrease associated with the impaired outcome. The reason for negative weights for GAF is that higher value corresponds to better outcome.

**
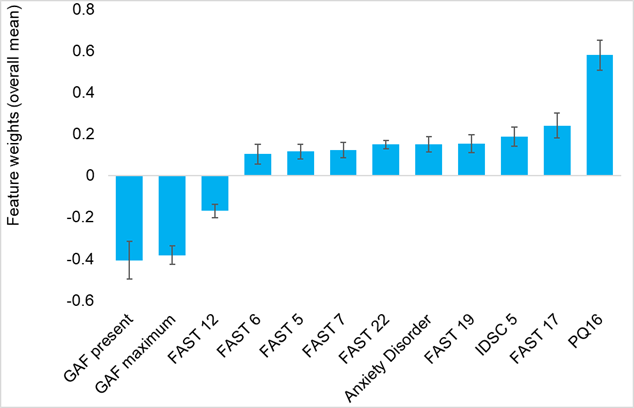
**

**Figure S4.** Time Series K-Means clustering implemented in Scikit-learn v. 1.5.2 to cluster the GAF trajectories in two clusters using 6 time points revealed a low- and high-functioning trajectory outcome. Subsequently, we used linear SVM to differentiate between the trajectory clusters using MRI features.


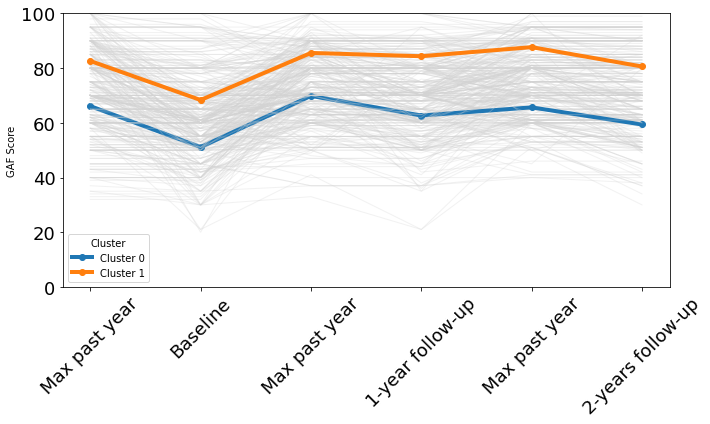


**Figure S5****.** Decision Curve Analysis (DCA), calculating net benefit across threshold probabilities and comparing each model with treat-all and treat-none strategies.


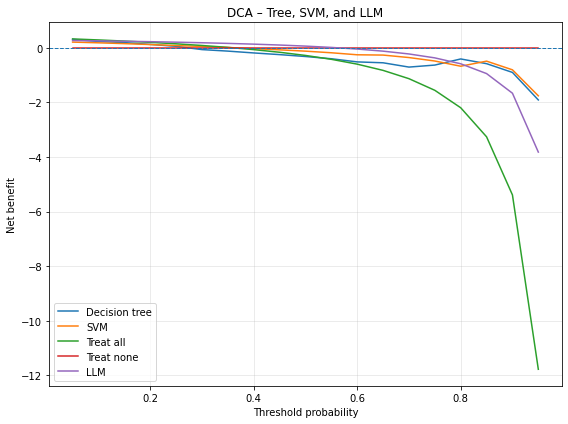


**Figure S6****.** Partial dependence plots (PDPs) of the top predictors for functional impairment. Higher partial dependence values (y-axis) correspond to a higher predicted probability of functional impairment. PDPs revealed that baseline and maximum GAF scores showed the strongest marginal effects, with marked decreases in predicted impairment once functioning exceeded approximately 55–60 (baseline) or 75–80 (maximum). Mood-related depressive symptoms (IDSC5) and functional difficulties in interpersonal (FAST19) and occupational domains (FAST7) also displayed positive relationships with predicted impairment, though with smaller effect sizes.


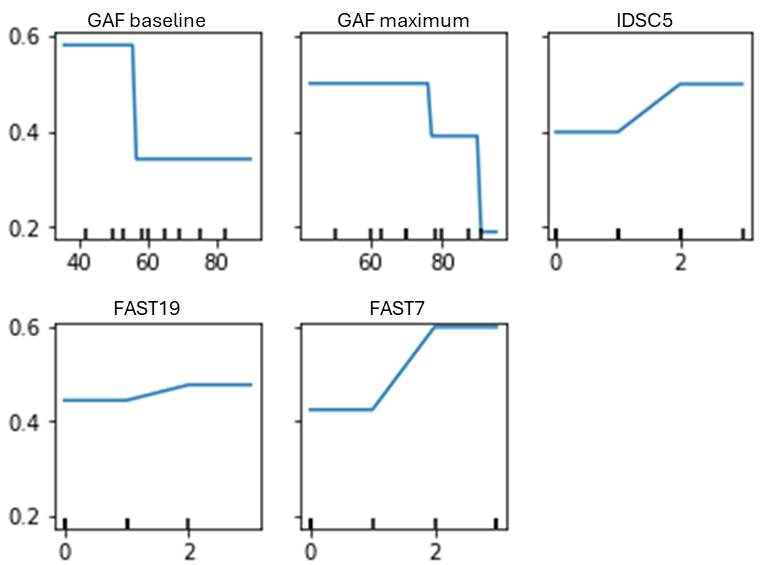

Supplement: Multimedia Appendix 1 [file mental-v13-e84424-s001.docx]
